# Instruments for Assessing Spirituality in Patients with Chronic or Advanced Illnesses: A Systematic Review of the Last 15 Years

**DOI:** 10.3390/healthcare14081013

**Published:** 2026-04-12

**Authors:** María Ángeles Portillo-Gil, Giancarlo Lucchetti, Rocío De Diego-Cordero

**Affiliations:** 1 Research Group “Comprehensive and Sustainable Health: A Bio-Psycho-Social, Cultural, and Spiritual Approach to Human Development—CTS-1149 (DesH-Global)”, School of Nursing, Physiotherapy and Podiatry, University of Seville, C./Avenzoar, 6, 41009 Seville, Spain; rdediego2@us.es; 2 Department of Medicine, School of Medicine, Federal University of Juiz de Fora, Juiz de Fora 36036-900, Brazil; g.lucchetti@yahoo.com.br

**Keywords:** spirituality assessment, palliative care, chronic illness, measurement instruments, psychometric evaluation

## Abstract

**Highlights:**

**What are the main findings?**

- This review identified 43 validated instruments measuring spirituality in adults with chronic or advanced illnesses or receiving palliative care, with most scales assessing cognitive, affective, and behavioral dimensions.
- Considerable variability was found in psychometric rigor, with limited evidence for criterion-related validity, especially predictive validity and frequent presence of potentially “contaminated” items overlapping with emotional or psychological constructs.

**What are the implications of the main findings?**

- Current spiritual assessment tools require clearer conceptual boundaries and culturally sensitive refinement to improve validity and reduce overlap with non-spiritual domains.
- Future development should prioritize longitudinal validation and clinically oriented instruments to enhance their applicability in routine healthcare and support more accurate evaluation of spiritual needs and outcomes.

**Abstract:**

**Background/Objectives**: Spirituality is a key component of coping and well-being in chronic and advanced illness, yet its assessment remains inconsistent across clinical settings. To identify, classify, and critically analyze the most commonly used and validated instruments for measuring spirituality in clinical contexts, focusing on their ability to assess the current spiritual state from a multidimensional perspective (cognitive, behavioral, and affective expressions). **Methods**: A systematic literature review was conducted using PubMed, Scopus, and Web of Science (2011–2024). Inclusion criteria targeted validation studies of instruments assessing spirituality in adults with chronic or advanced illnesses or in palliative care. A dual conceptual–functional classification was applied, and a custom scoring system was developed to evaluate psychometric quality. Contamination and tautological aspects were also examined. **Results**: Forty-three instruments were identified across 42 studies. Of these, 93.02% included cognitive, affective, and behavioral dimensions. Most were validated in oncology or chronic disease populations. Content validity and internal consistency were the most reported psychometric properties; responsiveness was rarely evaluated. **Conclusions**: The available instruments reflect several conceptual and functional approaches. The classification proposed in this review provides practical guidance for selecting scales according to specific clinical goals and settings, supporting the evaluation of the current spiritual state and the integration of spirituality into healthcare practice. Further research is recommended to develop culturally sensitive and responsive instruments suitable for diverse clinical contexts.

## 1. Introduction

In recent decades, multiple studies have explored the connection between spirituality and health in fields such as medicine, nursing, psychology, sociology, and theology [1]. Although initial research focused on religious aspects of healthcare, the growing secularization of societies has broadened interest toward a more inclusive concept of spirituality [2].

Clinical evidence consistently shows that spirituality can be a key resource for many patients when facing illness, contributing significantly to their quality of life, particularly in those with chronic or advanced conditions [3]. Conversely, low spiritual well-being or religious struggles have been associated with greater mortality, severe depression, hopelessness, and a desire to die. These findings have led health organizations to recommend the integration of spiritual aspects into clinical care [4].

Despite this, several challenges persist, including the absence of a universal definition of spirituality and the lack of agreement regarding its dimensions within the health field. This ambiguity has generated multiple interpretations of the construct and has complicated the selection and comparison of instruments used to measure it [5].

Although related, religiosity and spirituality are not equivalent. Koenig defines religiosity as the adherence to structured systems of beliefs, rituals, and symbols oriented toward the sacred [6]. In contrast, spirituality is understood as a personal search for meaning and answers to existential questions, as well as a relationship with the sacred or transcendent, with or without participation in organized religion [7]. Puchalski’s definition highlights spirituality as a dynamic, intrinsic aspect of humanity through which individuals seek meaning, purpose, and transcendence, expressed in relationships with oneself, others, society, nature, and the sacred [8]. The absence of a consensual definition has contributed to diverse conceptualizations of spirituality, generally grouped into two broad perspectives: a theistic perspective, centered on belief in God or a higher power, and a non-theistic perspective, focused on existential, humanistic, and secular dimensions of human experience [7].

The working definitions of the constructs of spirituality and religion on which this study was based appear in Table 1. These definitions served as the conceptual foundation for determining the eligibility of instruments, guiding both the classification of scales and the identification of items that reflect the current spiritual state.

To generate solid evidence in this field, precise and validated measurement instruments are essential. However, it remains unclear whether existing tools adequately assess patients’ current spiritual beliefs, attitudes, and needs, or whether they can guide clinical interventions. These limitations hinder the development of a spirituality-integrated model of care [12].

Although previous reviews have examined spiritual and religious instruments [13,14], recent analyses specifically addressing updated psychometric properties in populations with chronic or advanced illnesses are lacking, despite the fact that these groups rely more frequently on spiritual and religious coping. Furthermore, earlier reviews did not examine instruments from a multidimensional perspective incorporating cognitive, affective, and behavioral domains. Considering these dimensions is crucial, as they represent the main ways in which spirituality is expressed in clinical practice: cognitive (beliefs, interpretations, and meaning attributed to illness and life), affective (emotions such as peace, hope, or existential suffering), and behavioral (actions and practices used to cope with illness). Together, these domains form the basis for evaluating the current spiritual state, a dynamic construct that fluctuates according to an individual’s clinical, emotional, and existential experience [13].

For the purposes of this review, “current spiritual state” was operationally defined as the individual’s present cognitive, affective, and behavioral spiritual experience, as reflected through validated items capturing spiritual beliefs, emotions, and practices in the here-and-now. Based on this definition, instruments were classified as (1) primary spirituality measures, in which spirituality constitutes the central construct of the scale, and (2) mixed psychosocial instruments, which include a spiritual dimension embedded within a broader psychosocial assessment. The inclusion of mixed instruments is justified because their spiritual subscales provide validated, clinically relevant indicators of current spiritual experience in populations with chronic and advanced illness [13,15].

Given the conceptual and methodological diversity among spirituality instruments, this review proposes an integrative framework combining psychometric analysis with a multidimensional functional classification. This approach allows clinicians and researchers to identify tools most suitable for assessing the current spiritual state and informing patient-centered interventions. Therefore, this systematic review aims to identify, classify, and critically analyze the most widely used and validated instruments for measuring spirituality in clinical contexts, with particular emphasis on their ability to assess the current spiritual state across cognitive, behavioral, and affective dimensions.

## 2. Materials and Methods

### 2.1. Protocol and Registration

This systematic review was conducted following the guidelines of the PRISMA (Preferred Reporting Items for Systematic Reviews and Meta-Analyses) 2020 statement. The review protocol was not registered in PROSPERO or any other database prior to conducting the study. However, the methodology was defined in advance, including clear inclusion and exclusion criteria, search strategies, and data extraction procedures, all of which were maintained without changes throughout the entire review process.

### 2.2. Search Strategies

Searches were conducted in PubMed, Scopus, and Web of Science from January 2011 to December 2024. No language restrictions were applied. The last search was performed on 15 December 2024. The search strategy was developed by the main author and reviewed by the research team. The complete search strings are provided in Table 2.

Studies were included when they assessed the validation and psychometric properties of spirituality scales or instruments used in healthcare. Mixed psychosocial instruments were also included when they contained a specific spiritual dimension that aligned with the operational definition of “current spiritual state.” These instruments do not assess spirituality as a whole but provide validated items that capture aspects of spiritual experience relevant to clinical assessment. Their inclusion allows identifying instruments already used in clinical practice that incorporate spiritual components within broader psychosocial evaluations, ensuring that the review reflects all validated tools capable of assessing the patient’s current spiritual state. The following articles were excluded:

1. Studies in which the instrument was applied to healthy individuals, adolescents, children, healthcare professionals or family members/caregivers. These population groups were excluded from the target population because their spiritual experiences differ substantially from those of patients with chronic or terminal illnesses. Although caregivers may also go through complex spiritual processes in the context of caregiving and illness, scientific literature shows that their spiritual needs, approaches, and meanings are not equivalent to or comparable with those of patients directly affected by illness [16,17].
2. Scales that assessed only religious aspects without considering the spiritual dimension.
3. Articles published outside the established date range (2011–2024). This timeframe was selected, aiming to complement the previous systematic review published by Monod [13]. Therefore, the present review presents an updated panorama of the current validated studies.
4. Studies including patients not diagnosed with a chronic or terminal illness, according to the definition of chronic disease established by the World Health Organization (WHO) and the definition of terminal illness by the Spanish Society of Palliative Care (SECPAL). According to the WHO, chronic diseases, also known as noncommunicable diseases, are long-duration conditions with slow progression, resulting from a combination of genetic, physiological, environmental, and behavioral factors, and include cardiovascular diseases, cancer, chronic respiratory diseases, diabetes, among others [18]. SECPAL, on the other hand, defines terminal illness as an advanced, progressive, and incurable condition, with no reasonable expectation of response to specific treatment, presenting multiple intense, multifactorial, and variable physical problems, with a significant emotional impact on the patient, family, and therapeutic team, and a life expectancy of less than six months [19].
5. Measurement instruments consisting of only one item.
6. Patients diagnosed with mental illness or significant cognitive impairment. This exclusion is based on the fact that evaluating the spiritual dimension requires adequate insight, understood as the capacity for awareness and understanding of one’s own spiritual experience. In individuals with psychiatric disorders or cognitive decline, this capacity may be compromised, which affects the validity and reliability of the instruments used to measure spirituality [20,21]. Given these considerations, specific adaptations are necessary for use in these populations [22]. Therefore, their inclusion could bias the results and compromise the methodological rigor of the study.
7. Studies that did not correspond to a scale validation.

The selected articles were then subjected to a full-text review to examine the spirituality measurement instruments in depth. In this phase, scales were excluded if: (1) they referred exclusively to elements of religiosity; (2) they only consisted of one item related to spirituality; (3) there was no evidence that the instrument had been used with clinical outcomes; (4) data on the instrument’s psychometric properties were unavailable; and (5) they were studies other than a scale validation.

Finally, scholars and researchers in the field of religion and spirituality were asked to identify any additional scales or questionnaires that met the inclusion and exclusion criteria established in the study.

### 2.3. Study Selection Process

Two reviewers independently screened titles and abstracts obtained from the searches. Full-text articles were assessed for eligibility according to predefined inclusion and exclusion criteria. Discrepancies were resolved through discussion until consensus was reached.

### 2.4. Data Extraction

Two reviewers independently extracted data using a structured data extraction sheet. Extracted variables included: study characteristics, population, country, instrument type, number of items, dimensions, psychometric results (reliability, validity), and classification of items (cognitive, affective, behavioral). Any disagreements were resolved by consensus.

For each instrument, the underlying dimensions of the spirituality construct were established, in addition to the objectives established for the instrument’s development. Data related to the psychometric properties were systematically recorded. When information on correlations with other instruments was available, this was extracted to assess criterion-related validity. Data on concurrent validity were also analyzed when available. Furthermore, studies in which spirituality measurement tools showed correlations in cross-sectional studies or were predictive in longitudinal research on health outcomes were included to analyze both concurrent and predictive validity.

Each instrument was evaluated using a scoring system specifically developed for this review and based on recognized standards for instrument development [5]. This system assesses six methodological domains: content validity, construct validity, criterion validity, internal consistency, test–retest reliability, and sample adequacy [23]. Each domain is rated dichotomously (0 = absent/insufficient/not reported; 1 = adequately described and methodologically acceptable), yielding a total score from 0 to 6. Higher scores indicate greater methodological rigor and stronger psychometric evidence. The operational definitions and scoring rules for each domain are detailed in Table 3. The results of this assessment for each included study are presented in the Section 3.

### 2.5. Quality Assessment

The methodological quality of the validation studies was assessed using a standardized approach based on well-established criteria for psychometric research. Because of the nature of these instrument validation studies, we examined several key areas in each one: (1) how clearly the study objectives and the instrument’s purpose were described; (2) whether the sample size was appropriate for psychometric analyses. In this review, no specific quantitative threshold was applied to determine whether the sample size used in each validation study was “adequate”, given the heterogeneity of the psychometric designs included. However, as a general indicator of sufficiency, we considered whether the authors provided an explicit methodological justification for the sample size or whether the sample size was consistent with widely accepted standards for factor analysis (e.g., approximately 5–10 participants per item, or ≥150 participants for exploratory or confirmatory factor analyses). This criterion was used solely to contextualize methodological quality and was not applied as an exclusion criterion; (3) whether the statistical methods used were suitable for validation, and (4) how thoroughly the psychometric properties were reported.

Two reviewers independently assessed the quality of each study. Any disagreements were resolved through discussion or, when necessary, consultation. The results of the quality assessment informed the interpretation of the findings but were not used as exclusion criteria, since the main goal was to provide a comprehensive overview of the available instruments. Studies with methodological limitations are noted in the Section 3 and Section 4.

### 2.6. Classification of Instruments for Assessing Spirituality

The development of the included instruments involved defining (a) the conceptual aspect of spirituality that the instrument seeks to assess and (b) the elements that operationalize the concept of spirituality in question. The term “development of the instruments included” does not refer to the authors of this review creating or modifying any scale. Rather, it refers to the development processes described in the original validation studies. This includes how the construct was defined, how the items were generated, the preliminary design of the instrument, and the initial validation steps reported by the original authors. This information was extracted exclusively to evaluate the psychometric quality of the existing instruments. In this study, a classification of measurement tools is proposed that follows the line of reasoning used in instrument development, as shown in Table 4.

### 2.7. Recovery of Instruments That Include Elements That Measure a “Current” Spiritual State

Based on the multidimensional framework described in the Introduction, instruments were categorized according to whether they assessed the current spiritual state through affective (A), behavioral (B), and cognitive (C) expressions. Two reviewers independently classified each instrument, and discrepancies were resolved by consensus. The complete classification indicating the presence of each functional dimension is presented in Table 5 and Table 6.

The initial agreement for the classification was excellent (Cohen’s Kappa 0.969). Divergences between the reviewers were discussed and resolved by consensus [28].

**Table 5:** Table of instruments assessing spirituality.

Instrument	Author and Date	Items and Measurement	Quality	Sample and Validation	Validation	Scale Contamination
Spiritual Needs Assessment Scale for Patients (SNAP).	Rashmi K. Sharma 2012 [29].	23 items, 4-point Likert scale.	A, B, C 6.	*N* = 47 outpatient cancer patients of diverse ethnicities and religions at Maimonides Cancer Center (Brooklyn, USA)	Cronbach’s alpha was 0.95. Test–retest correlation coefficient was 0.69.	NO
The Functional Assessment of Chronic Illness Therapy—Spiritual Well-being (FACIT-Sp) scale, Persian version,	Jafari et al. 2013 [30].	12 items, 5-point Likert scale.	A, B, C 5.	*N* = 153 patients undergoing cancer treatment. (Iran).	Cronbach’s α of 0.90	YES
Chinese version of the Spiritual Needs Questionnaire (SpNQ -Ch).	Büssing, A. et al., 2013 [31].	17 items, 5-point LIKERT scale.	A, B, C 5.	*N* = 168. Chinese patients with chronic diseases. Changhai Hospital of Traditional Medicine. Patients had cancer (63%), pain conditions (10%), or other chronic conditions (23%). China.	Cronbach’s alpha was not available.	NO
Arabic version of the FACIT-Sp scale.	Mark Lazenby et al., 2013 [32].	12 items, 5-point Likert scale.	A, B, C 4.	*N* = 205 Arabic-speaking participants with chronic diseases. Jordan.	Cronbach’s alpha value 0.72.	YES
SECPAL Spirituality Group (GES) Questionnaire.	Enric Benito et al., 2014 [33].	8 items, 5-point Likert scale.	A, B, C 4.	*N* = 108 palliative care patients from all over Spain.	Cronbach’s alpha value 0.72.	NO
Chinese version (C-SpIRIT).	Yu-Ling Lin et al., 2015 [34].	21 items. 5-point Likert scale.	A, B, C 6.	*N* = 260 participants from the oncology clinic of a medical center in southern Taiwan, using a convenience sampling technique.	Cronbach’s alpha coefficients ranged from 0.73 to 0.88.	NO
Thai Scale of Spiritual Well-being (SWBS),	Chaiviboontham et al., 2016 [35].	20 items, 6-point Likert scale. 3 factors: existential well-being, religious well-being, and peace.	A, B, C 4	*N* = 196 participants from three tertiary hospitals in Bangkok and suburban Thailand	Cronbach’s alpha values were 0.96, 0.94 and 0.93 for each scale respectively. Kaiser–Meyer–Olkin value was 0.94.	YES
Daily Spiritual Experiences Scale-Chinese (DSES-C).	Graciete Lo et al., 2016 [36].	16 items, 6-point Likert scale.	A, B, C 5.	*N* = 321 cancer patients. Participants were recruited from four community oncology practices in New York City.	The Cronbach’s alpha value was 0.94.	NO
The Spanish version of the FACIT-Sp scale.	Jiménez-Fonseca et al., 2017 [37].	12 items, 5-point Likert scale.	A, B, C 5.	*N* = 504 patients with resected non-metastatic cancer (Spain).	The omega reliability estimate is 0.874. It does not use Cronbach’s alpha, but instead uses omega values.	YES
Spiritual Well-Being Scale–Mandarin version (SWBS–M).	Woung-Ru Tang and Chen-Yi Kao, 2017 [38].	20 items, 6-point Likert scale.	A, B, C 4.	*N* = 243 cancer patients from five teaching hospitals across Taiwan.	Cronbach’s coefficient of 0.89.	YES
EORTC QLQ-SWB32 Spiritual Well-being Questionnaire Scales.	B. Vivat and T. Young, 2017 [39].	32 items, the 22 items grouped into the four rating scales (Relationships with Others, Relationships with Self, Relationship with Someone or Something Greater, and Existential) and the single item Relationship with God scale are rated using a four-point scale. The Global Spiritual Well-being (Global SWB) item uses a seven-point response/scoring scale.	A, B, C 5.	*N* = 104 patients. These participants were recruited from three cancer centers located in the two largest cities in Cyprus.	Relationship with others: 0.8 Relationship with oneself: 0.7 Relationship with someone or something bigger: 0.7 Existential: 0.8 Relationship with God: 1 Global Spiritual Well-being: 1	NO
Philippine version of the Spiritual Coping Strategies scale (SCS-F).	Jonas Preposi Cruz et al., 2017 [40].	20 items, 4-point Likert scale.	A, B, C 5.	*N* = 162 Filipino hemodialysis patients. Participants were recruited from two hospitals in La Union, Philippines.	Cronbach’s alpha for the total scale was 0.90	NO
Spiritual Needs Questionnaire (SpNQ) Farsi version.	Khadijeh Hatamipour et al., 2018 [41].	19 items, 4-point Likert scale.	A, B, C 4	*N* = 400 cancer patients from Tehran Cancer Institute (Iran).	Overall Cronbach’s alpha was 0.91. The correlation coefficient was 0.82.	NO
SHALOM Spiritual Well-Being Questionnaire.	O. Riklikiene, S. Kaseliene and J. Fisher 2018 [42].	20 items, 5-point Likert scale.	A, B, C 4.	*N* = 171 non-terminally ill cancer patients in a public hospital (Lithuania).	Cronbach’s alpha ranged from 0.578 to 0.949	NO
Spiritual Well-being Scale (SWS) Brazilian version, Religious Well-being (RW) and Existential Well-being (EW).	Tavares Gomes and Muniz da Silva Bezerra 2018 [43].	20 items, 6-point Likert scale.	A, B, C 3.	*N* = 174 patients hospitalized in the preoperative period, in the wards of a university hospital specializing in clinical and surgical cardiology (cardiac myocardial revascularization surgery, valve replacement or repair) (Brazil).	Cronbach’s alpha of 0.78 Kaiser–Meyer–Olkin (KMO) of 0.87	NO
The Chinese version of the Spiritual Needs Questionnaire in cancer patients (SpNQ-Ch-27),	Y. Zhao et al., 2019 [44].	27 items, 4-point Likert scale.	A, B, C 4.	*N* = 457 patients with chronic illnesses in the Xinjiang Hospital (China).	Cronbach’s alpha of 0.90.	NO
Adaptation of the Nurses’ Spiritual Therapeutics Scale (C-NSTS) in China.	Haiyan Xie et al., 2019 [45].	18 items, 4-point Likert scale.	A, B, C 5.	*N* = 153 cancer patients who were hospitalized at one of two prominent university-affiliated tertiary hospitals in Beijing, China.	Cronbach’s alpha: 0.88.	NO
Spirituality Instrument-27 (SpI-27)	Weathers, E et al., 2020 [46].	27 items, 6-point Likert scale.	A, B, C 5	*N* = 222 patients with chronic non-malignant diseases (chronic pain, arthritis, high blood pressure, endometriosis, diabetes, asthma, chronic fatigue syndrome, Crohn’s disease) or long-term (18–55 years). Germany and Switzerland.	Cronbach value of 0.904	NO
Psychometric validation of the Spiritual Well-Being Scale—Mandarin version (SWBS–M).	Panchan Thapanakulsuk et al., 2020 [47].	20 items, 5-point Likert scale.	A, B, C 5.	Pilot study for the internal consistency test: The sample consisted of 30 cancer patients. Exploratory factor analysis (EFA): 190 patients participated in this stage. Participants came from all regions of Thailand. Confirmatory factor analysis (CFA) and concurrent validity: 203 patients participated in this phase.	Cronbach value: 0.76.	YES
The EORTC QLQ-SWB32 spiritual well-being assessment scale.	Kyranou and Nicolaou. 2021 [48].	32 items, most on a 4-point Likert scale, one on a 7-point Likert scale.	A, C 5	*N* = 451 patients from 14 countries on 4 continents and in 10 languages. Patients with cancer (lung, breast, colorectal, etc) receiving palliative care were in day centers and inpatient units, outpatient clinics, hospital wards, and at home.	Cronbach’s alpha of 0.8, intraclass correlation index greater than 0.7	NO
The Spiritual Well-being Scale for Functional Assessment of Chronic Illness Therapy (FACIT-Sp-12) Dutch version.	Damen et al., 2021 [49].	12 items, 5-point Likert scale.	A, B, C 4.	*N* = 400 Dutch patients with advanced cancer (stage IV). Netherlands.	Total Cronbach’s alpha of 0.82.	YES
Spiritual Distress and Resources Questionnaire (SDRQ).	Simon Peng-Keller et al., 2021 [50].	22 items, 6-point Likert scale.	A, B, C 5.	*N* = 219 patients with chronic pain. These patients were recruited from five different inpatient departments and outpatient clinics in the German-speaking part of Switzerland.	Cronbach’s alpha values: Spiritual Anguish: 0.93 (unchanged) Transcendence: 0.85 Immanence: 0.81 Spiritual coping (three items): 0.70	NO
Spiritual Distress Assessment Tool for Cancer Patients. Korean (SDAT)	Kim et al., 2022 [51].	20 items, 5-point Likert scale.	A, B, C 6.	*N* = 225 cancer patients. Sample of three institutions in Korea: Severance Hospital. Ulsan University Hospital. Seoul Medical Center	Reliability analysis confirmed high internal consistency with a Cronbach’s coefficient of 0.75 or higher.	NO
The FACIT-Sp spiritual well-being scale.	Ahmad et al., 2022 [52].	12 items, 5-point Likert scale.	A, B, C 4.	*N* = 200 subjects with serious or life-threatening illnesses (HIV, genetic diseases, etc.) recruited from the inpatient and outpatient clinics of the NIH Clinical Center (Bethesda, Maryland, USA).	Cronbach’s alpha 0.89.	YES
Spiritual Distress Scale (SpiDiScI).	Joris Gielen et al., 2022 [53].	16 binary items. The spiritual distress score is calculated by counting responses as 1 for sahmat/agree and 0 for asahmat/disagree.	A, B, C 4.	*N* = 400 cancer patients undergoing pain management in the pain and palliative care unit of a tertiary cancer hospital in New Delhi.	Cronbach’s alpha 0.85.	NO
Persian version of the SpREUK -P questionnaire.	Mehdi Pasalar et al., 2022 [54].	17 items, 4-point Likert scale.	A, B, C 4.	*N* = 233 adult patients with gastrointestinal disorders. Shahid Gastrointestinal Clinic Faghihi from Shiraz University of Medical Sciences (Shiraz, Iran).	Cronbach’s alpha 0.81 for the importance scale and 0.71 for the practices scale.	NO
Spiritual Well-being Questionnaire (EORTC QLQ-SWB32) (2022) Finnish version.	Goyarrola et al., 2023 [55].	32 items, 4-point Likert scale.	A, B, C 5.	*N* = 190 (101 cancer patients from oncology units and 89 patients with other chronic diseases from religious communities in different parts of the country). Finland.	Cronbach’s alpha 0.81	NO
The FACIT-Sp-12 Polish version.	Machul et al., 2023 [56].	12 items, 4-point Likert scale	C 4	*N* = 355 patients with chronic diseases (cancer, asthma, heart disease, arthritis, diabetes, and Crohn’s disease), admitted to a clinical hospital in eastern Poland.	Cronbach’s alpha was 0.614	YES
I-SPIRIT	Steinhauser et al., 2024 [57].	30 items, 5-point Likert scale.	A, B, C 5.	*N* = 249 ill patients (stage IV cancer, congestive heart failure, chronic obstructive pulmonary disease, end-stage renal disease) from the acute illness outpatient clinics of the Durham Veterans Affairs Healthcare System (UK).	Cronbach’s alpha ranges from 0.76–0.86 Intraclass correlation coefficients were 0.69–0.79	NO
Spiritual Instrument SpI-27 Turkish version.	Berfin Bingol and Medine Yılmaz, 2024 [58].	27 items, 5-point Likert scale.	A, B, C 5.	*N* = 267 patients who were hospitalized in a cardiology clinic and diagnosed with a chronic disease (Türkiye).	Cronbach’s alpha 0.927.	NO
Spiritual Wellbeing Scale (FACIT -Sp12).	Megan C. Best et al., 2024 [59].	12 items, 5-point Likert scale.	A, C 5.	*N* = 897 adult patients. Participants were recruited from six hospitals in Sydney, Australia.	Cronbach’s alpha for the total scale was 0.79.	YES
Persian version of the Spiritual Self-Care Practice Scale (SSCPS).	Asma Najmadini et al., 2024 [60].	23 items, 5-point Likert scale.	A, B, C 4.	*N* = 550 patients with cancer at various stages of the disease in Kerman, southeastern Iran.	Cronbach’s alpha value was 0.89.	NO
Questionnaire called S-SNAP (Sinhala version of the Spiritual Needs Assessment) Assessment for Patients).	Ramadasa et al., 2024 [61].	22 items, each domain is assessed by aggregating the numerical scores of the items that comprise it.	A, B, C 4.	*N* = 267 volunteers with cancer selected from three public cancer care institutions in Sri Lanka.	The overall Cronbach’s alpha for the S-SNAP was 0.94.	NO

A = Affective dimension; B = Behavioral dimension; C = Cognitive dimension. Scores range from 0 to 6, with higher scores indicating a more comprehensive validation process. “No” indicates that the instrument is not contaminated, and “Yes” indicates that the instrument is contaminated.

**Table 6:** Results. Scales containing at least one item assessing the spiritual dimension.

Instrument	Author and Date	Items and Measurement	Quality	Sample	Validation	Scale Contamination
The Quality of Life at the End of Life QUAL-E.	Christopher et al., 2011 [62].	26 items, 5-point Likert scale. QUAL-E Cancer reduced version 17 items, 5-point Likert scale.	A, B, C 5.	*N* = 464 patients with advanced cancer in palliative care with a life prognosis of 6 months to 2 years from 24 outpatient oncology clinics at Princess Margaret Hospital in Toronto, Canada.	Cronbach’s alpha of 0.81 for QUAL-E Cronbach’s alpha of 0.80 for QUAL-EC reduced version.	NO
Herth Hope Index (HHI).	Carla Ida Ripamonti et al., 2012 [63].	12 items, 4-point Likert scale. Strongly disagree. In disagreement (In disagreement) In accordo (In agreement) Strongly in agreement (Totally agree).	A, B, C 5.	*N* = 266 cancer patients from four different centers in Italy.	Cronbach’s alpha for the total scale was 0.84.	NO
Purpose in Life (PIL).	C. Brunelli et al., 2012 [64].	20-item, 7-point verbal scale with two anchoring statements.	A, B, C 5.	*N* = 266 cancer patients from Italian centers.	Cronbach’s coefficient Alpha was 0.91.	NO
Seeking Of Noetic Goals (SONG).	C. Brunelli et al., 2012 [64].	20 items, 7-point Likert scale.	A, B, C 5.	*N* = 266 cancer patients from Italian centers.	Cronbach’s coefficient alpha was 0.90.	NO
The World Health Organization quality of life HIV instrument (WHOQOL-HIV) version French.	Reychler et al., 2013 [65].	120 items and 37 questions, 5-point Likert scale.	A, B, C 5.	*N* = 52 French-speaking people with HIV (Belgium).	Cronbach’s alpha 0.70.	NO
QRFPC25.	Kouloulias et al., 2017 [66].	25 items, scale. Responses listed using a measurement scale with alphabetical and numerical measures, where each letter corresponded to a relative number.	A, B, C 5.	*N* = 156 patients of the Radiotherapy Unit (Aretaieion University Hospital), Radiology Department of the Medical School of Athens.	Cronbach’s alpha > 0.70.	NO
Italian version of Patient Dignity Inventory (PDI-IT).	Luigi Grassi et al., 2017 [67].	25 items, 5-point Likert scale.	A, B, C 5.	*N* = 194 patients with cancer, both advanced and non-advanced stages, who were outpatients at two Italian centers.	Cronbach’s alpha value was 0.96.	NO
German version of the QUAL-EC-Psychosocial Questionnaire (QUAL-EC-P).	Britta Grunke et al., 2018 [68].	14 items, 5-point Likert scale.	A, B, C 4.	*N* = 183 patients with an incurable advanced solid tumor. Patients were recruited from the University Medical Center Hamburg–Eppendorf and the University Medical Center Leipzig (Germany).	α = 0.77.	NO
Instrument for the Assessment of Psychosocial and Spiritual Needs of Patients at the End of Life (ENP-E).	Dolors Mateo-Ortega et al., 2019 [69].	14 items, 6-point Likert scale.	A, B, C 6.	*N* = 150 patients diagnosed with advanced cancer from 19 hospitals in Spain.	Cronbach’s alpha value for the ENP-E scale was 0.75.	NO
4-Dimensional Utrecht Symptom Diary (USD-4D).	De Vries et al., 2021 [70].	22 items, 11-point Likert scale.	A, B, C 5.	*N* = 12 patients with short life expectancy in a general hospital and a hospice.	No Cronbach’s alpha value is reported; this is a qualitative study.	NO

A = Affective dimension; B = Behavioral dimension; C = Cognitive dimension. Scores range from 0 to 6, with higher scores indicating a more comprehensive validation process. “No” indicates that the instrument is not contaminated, and “Yes” indicates that the instrument is contaminated.

### 2.8. Risk of Bias Assessment

The methodological quality and potential risk of bias of the included studies were evaluated through a structured psychometric appraisal system. Given that traditional risk of bias tools (e.g., Cochrane RoB) are designed for interventional studies and not for instrument validation, we applied a specific scoring framework (0–6) based on recognized standards for health measurement instruments. This system allowed us to systematically quantify the certainty of the evidence by assessing six critical domains: content validity, construct validity (CFA), criterion validity, internal consistency, test–retest reliability, and sample adequacy (Table 3). This multidimensional approach ensures that the findings reported for each scale are supported by a rigorous validation process, identifying any potential bias related to small sample sizes or incomplete psychometric reporting. Additionally, a comprehensive search across three major databases with no language restrictions was performed to minimize publication and selection bias.

## 3. Results

**Figure 1:**
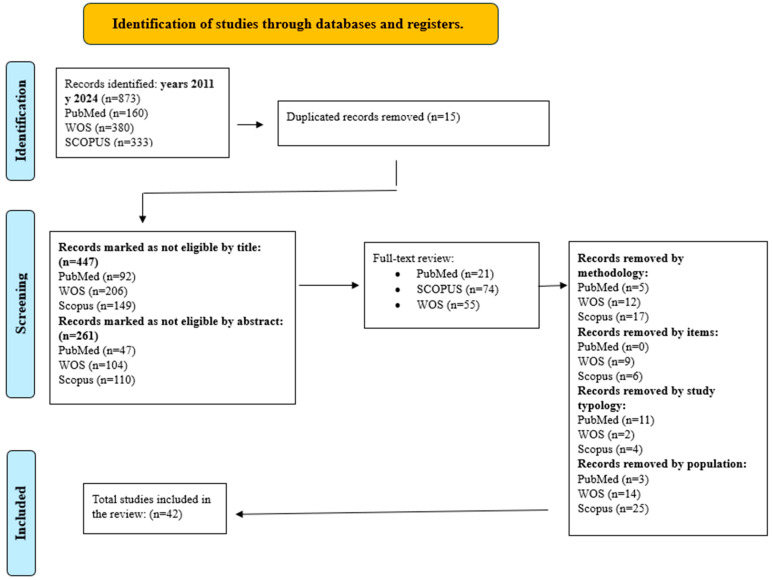
PRISMA flow diagram of the literature search and study selection process.

A total of 881 articles were identified through searches in PubMed, Scopus, and Web of Science, from which 42 studies were selected that validated 43 instruments for measuring spirituality in clinical settings. Some instruments had abbreviated versions and were validated in different countries, which were considered independent scales. The article selection process is detailed in Figure 1.

To facilitate a clearer understanding of the findings, the results are presented in a structured manner, beginning with the identification and classification of instruments, followed by their psychometric properties, and concluding with an analysis of dimensional patterns across scales.

### 3.1. Instruments to Assess Spirituality

Table 5 shows scales that include at least one item assessing the spiritual dimension. Among others, it shows the correlations observed between these spirituality measures and health outcomes in cross-sectional studies, reflecting their concurrent validity, and available data from prospective studies that analyzed the ability of these instruments to predict health outcomes, demonstrating their predictive validity.

As explained in the Section 2, mixed psychosocial instruments were included when they contained a validated spiritual domain relevant to assessing the current spiritual state. Table 6 presents mixed psychosocial instruments that include at least one validated spiritual item relevant to the assessment of the current spiritual state.

### 3.2. Quality of Studies Included

The methodological quality of the 43 identified spiritual assessment scales demonstrated a high level of rigor in their development process. All studies (*n* = 42, 100%) clearly stated their objectives and described the instrument purpose. Sample sizes ranged from 12 to 897 participants (median = 225), with the majority (*n* = 40, 93.02%) meeting recommended minimum sample sizes for psychometric analysis (*n* ≥ 100).

Most scales reported a comprehensive psychometric evaluation. Content validity was the most frequently reported property (*n* = 42, 97.67%), followed by criterion validity (*n* = 41, 95.35%) and construct validity through factor analysis (*n* = 41, 95.35%). Internal consistency (reliability) was established for 93.02% of the scales (*n* = 40). However, responsiveness to change (*n* = 4, 9.3%) was the only property with significantly lower reporting, due to the cross-sectional design of most studies.

Transparency in reporting was generally adequate, with most studies providing sufficient detail about methods and acknowledging limitations. The quality assessment results are summarized in Table 7. Overall, while the included studies demonstrated acceptable methodological rigor, there was considerable variability in the comprehensiveness of psychometric evaluation, which should be considered when interpreting the findings.

### 3.3. Sample for Validation

The instrument validated with a larger sample of participants is the Spiritual Wellbeing Scale (FACIT Sp12), which was studied with 897 individuals in Sydney, Australia. Overall, the samples included in the reviewed studies consisted of 64.29% cancer patients; 28.57% patients with chronic diseases such as diabetes or respiratory, cardiac, or renal diseases, and 7.14% consist of studies with a mixed population. The instrument developed with the greatest variability in the sample was the EORTC quality of life, with a sample size of 451 collected in 14 different countries. Five instruments were validated in different populations, and since one of them met the exclusion criteria (such as healthy individuals), it was excluded due to the impossibility of disaggregating the data. 

### 3.4. Psychometric Properties

The following subset comprises studies offering the most impactful data for clinical application.

The validation study of the Lithuanian version of SHALOM cites as a limitation the need for evidence of its concurrent validity, comparing the findings with data from other sources and applying congruent or divergent measurement tools. Concurrent validity is a type of criterion-related validity.

In the validation study of the Psychosocial and Spiritual Needs Assessment Instrument for Patients at the End of Life (ENP-E), criterion-related validity was established by correlating the ENP-E scale with other instruments measuring emotional well-being, such as the Hospital Anxiety and Depression Scale (HADS), the Distress Thermometer (DT), and the Quality of Life item (QLQ-15) from the EORTC QLQ-C15-PAL. A positive correlation was observed with the HADS and DT, and a negative correlation with the QLQ-15, supporting the instrument’s sensitivity to emotional and quality-of-life dimensions in end-of-life contexts.

Similarly, in the validation of the I-SPIRIT scale, convergent and discriminant validity were assessed by comparing the instrument with the FACIT-Sp, the BMMRS, the POMS, the PHQ-8, and the physical and social/family well-being subscales of the FACT-G. The correlations between the I-SPIRIT factors (Spiritual Needs and Spiritual Resources) and these comparator measures provided evidence for the instrument’s alignment with related constructs (convergent validity) and its distinction from unrelated domains (discriminant validity).

In contrast, the validation of the QRFPC questionnaire employed indirect methods, assessing validity by comparing scale scores across patients with varying levels of functional status as measured by the ECOG scale. While this approach linked the instrument’s scores to a clinically relevant variable, it does not constitute a traditional criterion validity assessment involving comparison with an established “gold standard.”

The Arabic version of the FACIT-Sp states that the concurrent validity of the FACIT-Sp has been well established in previous studies and that its results are comparable. Pearson correlation coefficients were calculated to estimate the shared variance between the FACT-G subscales and the Spiritual Well-being factors, providing evidence of convergent validity.

Regarding predictive validity, we can state that it was scarce since most of the research had cross-sectional designs.

### 3.5. Classification of the Instruments

Spirituality measurement instruments are often multidimensional and have diverse objectives, such as assessing expressions of spirituality, beliefs, or spiritual experiences. The instruments were classified as measures of spiritual well-being; spiritual resources and coping or support; spiritual needs; professional sensitivity and competence; spiritual experiences; spiritual self-care practices; and as measures of spiritual perspective and related activities.

The established classification is based on the definition of three categories of items (cognitive, behavioral, and affective), according to the spiritual expression they attempt to represent. All of the instruments include items that investigate cognitive aspects, 42 of the 43 scales measure affective aspects of spirituality, and 40 of the 43 scales analyze behavioral aspects of spirituality. Overall, 93.02% represented all three categories together.

### 3.6. Items of the Measuring Instruments

**Figure 2:**
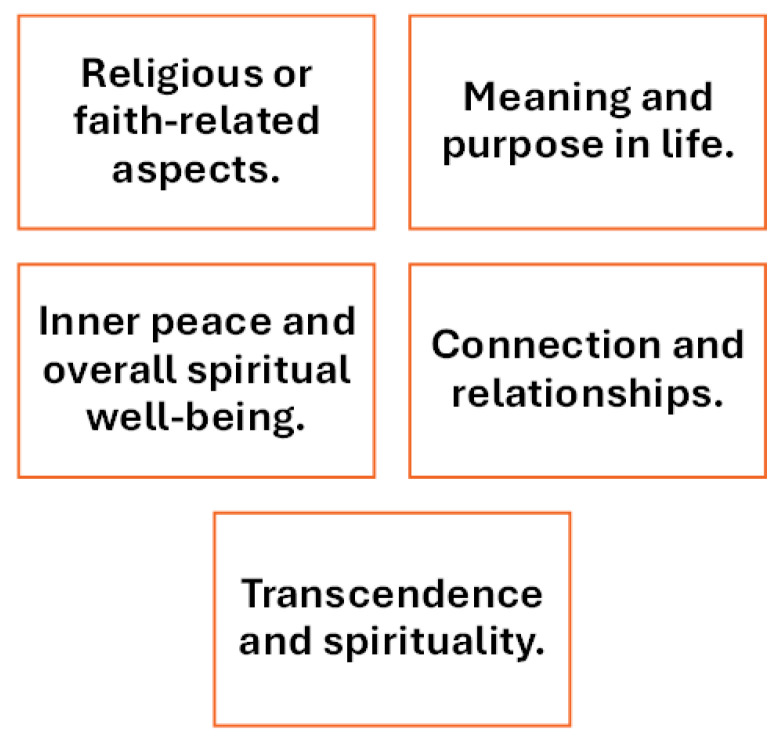
Prevalent dimensions of spirituality scales included in the review.

The dimensions that are most repeated among the scales analyzed can be grouped into the following main concepts represented in Figure 2.

Although these concepts are often used, the way they are defined and measured can vary significantly across scales, reflecting the diverse theoretical and cultural approaches to spirituality.

**Figure 3:**
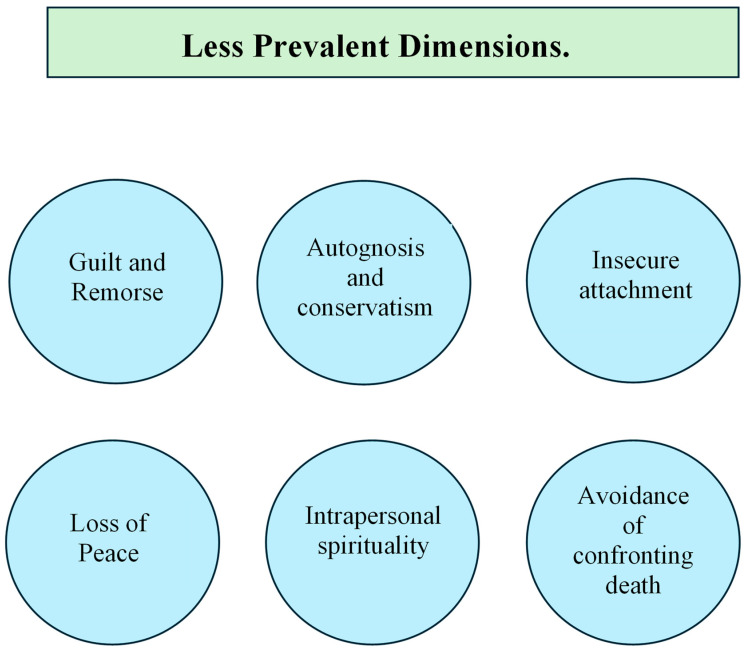
Less frequently represented dimensions in spirituality measurement scales.

Some of these dimensions that are least repeated between the scales are represented in Figure 3.

Focusing on these underrepresented dimensions is crucial, as they capture clinically significant nuances of spiritual distress that frequently surface in patients facing chronic or advanced stages of illness. Factors such as guilt, a loss of inner peace, or the avoidance of thoughts regarding death are well-established predictors of emotional suffering and poor adjustment to disease; nonetheless, they remain largely overlooked by current assessment scales. Similarly, constructs like insecure attachment or intrapersonal spirituality point to deeper identity and relational processes that shape how a patient interprets their situation and manages uncertainty. The scarcity of these indicators in existing tools suggests that many instruments might be missing critical facets of the current spiritual state, thereby hindering the ability to identify specific needs or sources of distress essential for tailored spiritual care.

### 3.7. Scales with Possible Conceptual Contamination

During the analysis of the content and structure of the instruments included in this review, it was identified that several of the scales commonly used to measure spirituality present elements that could be conceptually contaminated; that is, they may suffer from possible tautological problems, including items that overlap with dimensions of psychological, emotional, or social well-being. According to recent literature [71], this type of overlap may compromise the discriminant validity of the instruments and can induce tautological associations in correlational studies with mental health. Therefore, highlighting these problems could serve as a point of attention to those interested in the area.

Among the instruments analyzed in this study, the following scales have been previously reported as contaminated:

- FACIT-Sp: includes items related to meaning, purpose, inner harmony and comfort.
- WHOQOL-SRPB: integrates dimensions of mental health, quality of life and social connection.
- SWBS and SWBQ: potentially contaminated when existential and religious subscales are combined.
- SIWB: incorporates elements associated with life purpose and emotional well-being.
- STS: Includes indicators of inner peace, connection, and emotional transcendence.

## 4. Discussion

This systematic review aimed to identify and analyze available scales for assessing current spiritual status specifically in adult patients receiving palliative care and adult patients with chronic and advanced illnesses, examining the psychometric properties and validity of these instruments from a three-dimensional perspective (cognitive, behavioral, and affective) to understand how spirituality is measured in these relevant clinical contexts.

The identification of a significant number of scales validated in diverse languages and cultures (as evidenced by studies in cancer patients [41,44], and in patients with other chronic diseases [46,58]) underlines the growing attention toward the assessment of spirituality in these specific health domains.

When analyzing the psychometric properties of the identified scales, considerable variability in their reliability was observed. For example, the Italian version of the Patient Dignity Inventory (PDI-IT) [67] demonstrated excellent internal consistency with a Cronbach’s alpha of 0.96. Similarly, the Farsi version of the Spiritual Needs Questionnaire (SpNQ) [41] reported a Cronbach’s alpha of 0.91 in a sample of 400 cancer patients. These findings suggest adequate reliability in specific populations. However, other scales showed more modest internal consistency, such as the Polish version of the FACIT-Sp-12 [56], with a Cronbach’s alpha of 0.614 in patients with various chronic diseases. This variability underlines the importance of considering the reliability reported for the population and the context in which the scale is intended to be used, as emphasized by Sánchez and Echeverry [27].

It is important to clarify that Cronbach’s alpha represents an indicator of internal consistency reliability only. Some of the original validation studies interpreted high alpha coefficients as indirect support for construct coherence, but such values do not constitute evidence of validity. In this review, Cronbach’s alpha was considered exclusively as a measure of reliability, while validity was assessed through distinct psychometric approaches such as factorial analyses or criterion-related evaluations.

Regarding validity, most studies focused on construct validity (through exploratory or confirmatory factor analysis) and internal consistency. For example, the Spanish version of the Functional Assessment of Chronic Illness Therapy–Spiritual Well-Being Scale (FACIT-Sp) [37] demonstrated an acceptable level of reliability, as indicated by McDonald’s Omega coefficient, and its factorial structure was examined in oncology patients. Similarly, the Turkish version of the Spirituality Instrument-27 (SpI-27) [58] showed a Cronbach’s alpha of 0.927 in cardiology patients, and their study also explored the construct validity. However, an important limitation, consistent with what was reported in the review by Bhagwandas et al. [72], is the relative scarcity of studies assessing criterion-related validity, especially predictive validity. The ability of these scales to predict relevant health outcomes over time remains an open question in many cases.

The diversity of scales identified in this review reflects the complexity and multidimensional nature of the spirituality construct in these specific contexts. The lack of a single, universally accepted definition of spirituality [6] is manifested in the different approaches taken by the scales we analyzed. The dimensionality of spirituality remains a complex issue, as illustrated by the factorial instability of the SWBS [38] and the different structures found for the SpNQ in its 27-item Chinese version [44] compared to the original version. The existence of six dimensions in the SpNQ-Ch-27, influenced by Chinese cultural factors such as avoidance of discussion about death, highlights how cultural factors can shape the expression of spiritual needs. The suggestion to differentiate between the existential and religious aspects of spirituality, based on the study of HHI in patients with non-advanced cancer [63], also contributes to this debate.

In contrast to general spirituality scales, instruments specifically designed to assess spiritual needs (such as the SpNQ, SNAP or I-SPIRIT) demonstrate greater clinical usefulness. These scales include items that identify concrete areas of spiritual suffering, available resources and unmet needs, allowing clinicians to guide individualized interventions and support clinical decision-making in palliative care and chronic illness. Consequently, their clinical value is higher than that of global spirituality scales, whose purpose is more descriptive than diagnostic.

This dimensionality could explain the paradox of the existence of many scales for measuring spirituality and their underuse in clinical practice. This contradiction can be understood by another series of interrelated factors evidenced in the literature, such as methodological and psychometric issues, since identified problems of validity, reliability, and cultural bias in many of the tools reviewed. These methodological deficiencies can undermine health professionals’ confidence in the usefulness of the scales for clinical decision-making. Another reason is the disconnect between the scales and clinical needs, since Monod et al. [13] observed that many spirituality scales have been developed primarily for research purposes and are not designed for use in daily clinical practice. Health professionals may perceive that the existing scales are too long, complex, or irrelevant to their clinical needs, which hinders their adoption in practice. Finally, contextual and practical barriers such as lack of training of health professionals in spiritual assessment, cultural differences in understanding spirituality, and conflicting clinical priorities may also contribute to the underutilization of the scale by Selman et al. [15].

The concern about “contaminating elements” in spirituality scales [71] is also relevant in our populations of interest. It is crucial to consider whether items on scales used with patients with chronic illnesses or in palliative care might be influenced by their physical or mental health status (such as depression or fatigue), which could artificially inflate or deflate spiritual well-being scores.

### 4.1. Limitations and Strengths

This study presents limitations such as the heterogeneity of the validated samples, which makes direct comparison of results across studies difficult and limits the ability to draw universal conclusions about the psychometric properties of spirituality scales. Furthermore, it suggests that the validity and reliability of the scales may vary significantly depending on the specific population, highlighting the need for caution when selecting and applying these instruments in clinical practice and research. Furthermore, we found a predominance of cross-sectional studies. This type of design, although useful for exploring associations between variables at a given time, does not allow for establishing causal relationships or examining the evolution of spirituality over time [3]. Our findings confirm this limitation, as only 9.3% of the included studies provided evidence of responsiveness to change. In the context of spirituality and health, it is crucial to understand how spirituality influences health outcomes over time and how these outcomes, in turn, may affect the individual’s spirituality. In the specific context of chronic and advanced illness, where spirituality often fluctuates in response to suffering or clinical interventions, this lack of longitudinal validation and sensitivity data represents a major barrier for the integration of these tools into routine clinical follow-up. Finally, the presence of contaminated items may compromise the discriminant validity of the scales, that is, their ability to measure spirituality as a construct distinct from other aspects of well-being. This can lead to tautological associations in correlational studies, where the high correlation between spirituality and mental well-being is partly due to the scales measuring similar constructs.

This study also has clear strengths. First, a systematic and structured search was conducted using several databases and complemented by input from experts in the field. Furthermore, the proposed functional classification was validated based on the triple abstraction process conducted by blinded reviewers. Excellent agreement was observed. Additional data from subsequent studies using these instruments (e.g., data on concurrent and predictive validity) were systematically retrieved from the search. Finally, this review was not limited to English-language instruments but also included some measures initially developed in French, German, and Korean.

This review may also be limited by potential publication bias, as unpublished validation studies or non-indexed instruments may not have been identified. In addition, the exclusion of studies not published after 2011 may have led to the omission of earlier but still relevant validation work.

### 4.2. Recommendations for Future Research

Given the identification of “contamination” in several spirituality scales, further research is needed to develop and validate scales that minimize this problem and more accurately capture the unique dimensions of spirituality. This entails an effort to refine the conceptual definition of spirituality and to identify items that more purely reflect its essential components.

Developing more discriminating scales may require the use of qualitative research methods to explore the experience of spirituality in depth in different populations and the application of advanced statistical techniques, such as confirmatory factor analysis, to validate the structure of the scales and ensure that they measure distinct constructs.

Given the limitations imposed by the predominance of cross-sectional studies, it is recommended that future research adopt longitudinal designs, as they allow for the examination of the trajectory of spirituality over time. This is especially relevant in clinical populations where spirituality can fluctuate in response to illness, treatment, and other factors. By conducting repeated measurements of spirituality and health outcomes in the same individuals over time, researchers can identify patterns of change, determine the direction of relationships between variables, and assess the predictive capacity of spirituality in relation to clinical outcomes.

## 5. Conclusions

This systematic review provides an overview of the current landscape of spirituality assessment scales relevant to patients with chronic illnesses and palliative care. While various instruments are available, their psychometric quality and validity vary significantly. The identification of potential “contaminating elements” in some widely used scales underscores the need for a cautious interpretation of results and the development of more rigorous and conceptually clear tools. Future research should focus on strengthening the psychometric properties of the scales, especially responsiveness to change, predictive validity, and addressing conceptual challenges to advance our understanding of the role of spirituality in the context of health and healthcare.

To advance this field, future studies should prioritize methodologically rigorous validation of spirituality scales, with a focus on clearly defined constructs, hypothesis testing, and the use of longitudinal designs.

Addressing these limitations will improve the accuracy and usefulness of spirituality measures in assessing spiritual needs and evaluating the effectiveness of spiritual interventions.

## Figures and Tables

**Table 1:** Conceptual definitions used in this study.

Spirituality: Spirituality is recognized as a contributing factor to health in many people. The concept of spirituality is found in all cultures and societies. It is expressed in the individual search for ultimate meaning through participation in religion and/or belief in God, family, naturalism, rationalism, humanism, and the arts. Furthermore, it is considered as the part of people that finds coherence, meaning, and purpose in their lives. All of these factors can influence how patients and health professionals perceive health and illness and how they interact with each other [9]. “Spirituality is the dynamic dimension of human life that relates to the way people (individuals and community) experience, express and/or search for meaning, purpose and transcendence, and the way they connect with the moment, with themselves, with others, with nature, with the meaningful and/or the sacred” [8].
Religion: An organized system of beliefs, practices, rituals, and symbols designed to facilitate closeness to the sacred or transcendent (God, higher power, or ultimate truth/reality), fostering an understanding of the relationship and responsibility of each person toward others when living together in community [6].
Spiritual health or spiritual well-being: A person’s well-being arising from satisfaction or dissatisfaction with the areas of life that are important to them. It can refer to a vertical dimension, referring to a sense of a satisfying relationship with a higher or divine being; or to a horizontal dimension, referring to spiritual well-being derived from the meaning and purpose of life [10].
Spiritual Needs: Spiritual needs include, among other things, the ability to express one’s concept of God, deity, or divinity; to gain a sense of meaning and purpose; to express personal beliefs and values; to engage in spiritual practices; to experience love and harmonious relationships; to develop a greater sense of trust; to identify a source of hope and strength; and to receive and give forgiveness [11].

**Table 2:** Database search strategies and parameters.

Database	Date of Last Search	Search String	Filters/Operators
PubMed	15 December 2024	((“spiritual”[All Fields] OR “spiritualism”[MeSH Terms] OR “spiritualism”[All Fields] OR “spirituality”[MeSH Terms] OR “spirituality”[All Fields] OR “spiritualities”[All Fields] OR “spirituality s”[All Fields] OR “spiritually”[All Fields] OR “spirituals”[All Fields]) AND (“scale s”[All Fields] OR “scaled”[All Fields] OR “scaling”[All Fields] OR “scalings “[All Fields] OR “weights and measures”[MeSH Terms] OR (“weights”[All Fields] AND “measures”[All Fields]) OR “weights and measures”[All Fields] OR “scale”[All Fields] OR “scales”[All Fields])).	No language restrictions. Operators: OR, AND.
Web of Science	15 December 2024	((“spirituality” OR “spiritual well-being”) AND (“scale” OR “instrument”)) AND ((“adult” OR “hospitalized” OR “inpatient”) OR “palliative care”) AND (reliability OR validity)	Filters: Date (2011–2024) and Adult. Operators: OR, AND.
SCOPUS	15 December 2024	((“spirituality” OR “spiritual well-being”) AND (“scale” OR “instrument”)) AND ((“adult” OR “hospitalized” OR “inpatient”) OR “palliative care”) AND (reliability OR validity) “spirituality scale” AND (validation OR reliability).	Filters: Date (2011–2024) and Adult. Operators: OR, AND. No filters. Operators: OR, AND.

**Table 3:** Definitions and types of reliability and validity in measurement instruments.

Reliability: Refers to the stability and consistency of a measurement. It includes test–retest reliability, which assesses the stability of results at different times, and internal consistency, which analyzes how accurately the items in an instrument reflect the same concept, generally using Cronbach’s alpha coefficient. Test–Retest Reliability (Temporal Stability): The instrument’s ability to yield consistent results within the same sample across two different points in time, assuming the construct (spirituality) remains unchanged. It is typically measured using the Intraclass Correlation Coefficient (ICC) [24].
Validity: Indicates whether an instrument actually measures what it claims to measure. It is divided into several categories: Content validity: Determines whether the items adequately represent the concept being investigated. Construct validity: Analyzes whether the measurement meets theoretical expectations, using factor analysis or other methods. Criterion validity: Evaluates the relationship of the instrument to an external measure considered a reference standard. It can be concurrent (comparison with another measure at the same time) or predictive (ability to anticipate future outcomes). Discriminant validity: Analyzes whether the instrument can adequately capture differences between different groups [25].

**Table 4:** Classification Based on Spirituality Expressions.

CONCEPTUAL CLASSIFICATION. Based on the latent concept of spirituality that the measurement instrument, according to the authors who developed it, seeks to capture. Four common measurement categories are defined: general spirituality, religion, well-being, and spiritual needs.
FUNCTIONAL CLASSIFICATION. This classification is developed from an analysis of all the elements covered by the instrument [26]. Three categories are proposed depending on the expression of spirituality they aim to measure: Cognitive expressions: these elements aim to measure attitudes and beliefs towards spirituality [24]. Affective expressions: these components aim to capture feelings related to spirituality. Behavioral expressions: public or private practices of spirituality [27].

**Table 7:** Methodological quality assessment and psychometric properties of the included instruments.

Article	Objective Clarity	Sample ≥ 100	Content Validity	Criterion Validity	Confirmatory Factor Analysis
Hatamipour et al. [41].	✓	✓	✓	✓	✓
Machul et al. [56].	✓	✓	✓	✓	✓
Chaiviboontham et al. [35].	✓	✓	✓	✓	✓
Y. Zhao et al. [44].	✓	✓	✓	⮾	✓
Kyranou and Nicolaou [48].	✓	✓	✓	✓	⮾
Rashmi K [29].	✓	⮾	✓	✓	⮾
Steinhauser et al. [57].	✓	✓	✓	✓	✓
Kim et al. [51].	✓	✓	✓	✓	✓
Weathers et al. [46].	✓	✓	✓	✓	✓
Berfin Bingol and Medine Yılmaz [58].	✓	✓	✓	⮾	✓
Goyarrola et al. [55].	✓	✓	✓	✓	✓
Ahmad et al. [52].	✓	✓	✓	✓	✓
Jiménes-Fonseca et al. [37].	✓	✓	✓	✓	✓
O. Riklikiene, S. Kaseliene and J. Fisher [42].	✓	✓	✓	✓	✓
Jafari et al. [30].	✓	✓	✓	✓	✓
Tavares Gomes and Muniz da Silva [43].	✓	✓	⮾	✓	✓
Damen et al. [49].	✓	✓	✓	✓	✓
Joris Gielen et al. [53].	✓	✓	✓	✓	✓
Büssing, A et al. [31].	✓	✓	✓	✓	✓
Benito et al. [33].	✓	✓	✓	✓	✓
Lin et al. [34].	✓	✓	✓	✓	✓
Peng-Keller et al. [50].	✓	✓	✓	✓	✓
Graciele Lo et al. [36].	✓	✓	✓	✓	✓
C. Best et al. [59].	✓	✓	✓	✓	✓
Haiyan Xie et al. [45].	✓	✓	✓	✓	✓
Najmadini et al. [60].	✓	✓	✓	✓	✓
Pasalar et al. [54].	✓	✓	✓	✓	✓
Woung-Ru Tang and Chen-Yi Kao [38].	✓	✓	✓	✓	✓
Vivat and Young [39].	✓	✓	✓	✓	✓
Ramadasa et al. [61].	✓	✓	✓	✓	✓
Preposi Cruz et al. [40].	✓	✓	✓	✓	✓
Lazenby et al. [32].	✓	✓	✓	✓	✓
Thapanakulsuk et al. [47].	✓	✓	✓	✓	✓
Reychler et al. [65].	✓	⮾	✓	✓	✓
Christopher et al. [62].	✓	✓	✓	✓	✓
Vries et al. [70].	✓	⮾	✓	✓	Not reported
Britta Grunke et al. [68].	✓	✓	✓	✓	✓
Kouloulias et al. [66].	✓	✓	✓	✓	✓
Mateo-Ortega et al. [69].	✓	✓	✓	✓	✓
Grassi et al. [67].	✓	✓	✓	✓	✓
Ripamonti et al. [63].	✓	✓	✓	✓	✓
Brunelli et al. (PIL) [64].	✓	✓	✓	✓	✓
Brunelli et al. (SONG) [64].	✓	✓	✓	✓	✓

Note: Sample: Minimum required for psychometric stability (*N* ≥ 100). A checkmark (✓) indicates that the criterion was successfully met, including a minimum sample size requirement of *N* ≥ 100 for statistical robustness in factor analysis and reliability testing. A cross (⮾) indicates that the criterion was not met or the sample size was insufficient.

## Data Availability

No new data were created or analyzed in this study. Data sharing is not applicable to this article.
